# Study protocol P-MAPS: microbiome as predictor of severity in acute pancreatitis—a prospective multicentre translational study

**DOI:** 10.1186/s12876-021-01885-4

**Published:** 2021-07-31

**Authors:** C. Ammer-Herrmenau, T. Asendorf, G. Beyer, S. M. Buchholz, S. Cameron, M. Damm, F. Frost, R. Henker, R. Jaster, V. Phillip, M. Placzek, C. Ratei, S. Sirtl, T. van den Berg, M. J. Weingarten, J. Woitalla, J. Mayerle, V. Ellenrieder, A. Neesse

**Affiliations:** 1 Department of Gastroenterology, Gastrointestinal Oncology and Endocrinology, University Medical Center, Robert-Kochsstraße 40, 37075 Göttingen, Germany; 2 Department of Medical Statistics, University Medical Center, Göttingen, Germany; 3 Department of Medicine II, University Hospital, LMU Munich, Munich, Germany; 4 Department of Medicine I, University Hospital Halle, Halle, Germany; 5 Department of Medicine A, University Medicine Greifswald, Greifswald, Germany; 6 Division of Gastroenterology, Medical Department II, University Hospital of Leipzig, Leipzig, Germany; 7 Department of Medicine II, University Hospital Rostock, Rostock, Germany; 8 Department of Medicine II, University Hospital rechts der Isar, Technical University Munich, Munich, Germany; 9 Department of Medical Bioinformatics, University Medical Center, Göttingen, Germany

**Keywords:** Acute pancreatitis, Biomarker, Orointestinale microbiome, Metagenomic sequencing, Multicentric, Oxford nanopore technologies, ONT, P-MAPS, Prospective, Severity, NCT04777812

## Abstract

**Background:**

Acute pancreatitis (AP) is an inflammatory disorder that causes a considerable economic health burden. While the overall mortality is low, around 20% of patients have a complicated course of disease resulting in increased morbidity and mortality. There is an emerging body of evidence that the microbiome exerts a crucial impact on the pathophysiology and course of AP. For several decades multiple clinical and laboratory parameters have been evaluated, and complex scoring systems were developed to predict the clinical course of AP upon admission. However, the majority of scoring systems are determined after several days and achieve a sensitivity around 70% for early prediction of severe AP. Thus, continued efforts are required to investigate reliable biomarkers for the early prediction of severity in order to guide early clinical management of AP patients.

**Methods:**

We designed a multi-center, prospective clinical-translational study to test whether the orointestinal microbiome may serve as novel early predictor of the course, severity and outcome of patients with AP. We will recruit 400 AP patients and obtain buccal and rectal swabs within 72 h of admission to the hospital. Following DNA extraction, microbiome analysis will be performed using 3rd generation sequencing Oxford Nanopore Technologies (ONT) for 16S rRNA and metagenomic sequencing. Alpha- and beta-diversity will be determined and correlated to the revised Atlanta classification and additional clinical outcome parameters such as the length of hospital stay, number and type of complications, number of interventions and 30-day mortality.

**Discussion:**

If AP patients show a distinct orointestinal microbiome dependent on the severity and course of the disease, microbiome sequencing could rapidly be implemented in the early clinical management of AP patients in the future.

*Trial registration*: ClinicalTrials.gov Identifier: NCT04777812

## Background

Acute pancreatitis (AP) is an inflammatory disorder that causes a considerable economic health burden [1]. While the majority of AP show a mild clinical course, about 20% of patients suffer from moderate or severe disease with major local and systemic complications and a prolonged hospital stay [2, 3]. Furthermore, severe pancreatitis is associated with a dramatic increase of mortality ranging between 36 and 50% [4–6]. The revised Atlanta classification is a feasible scoring system to distinguish between mild, moderately severe and severe pancreatitis [3]. However, the revised Atlanta classification is determined in retrospect and does not guide early clinical management and risk assessment. For several decades multiple clinical and laboratory parameters have been evaluated, and complex scoring systems were developed to predict the clinical course of AP upon admission. However, scoring systems such as the *Ranson score*, *Acute Physiology and Chronic Health Evaluation II score* (APACHE II), *Bedside Index for severity in acute pancreatitis* (BISAP), *Harmless acute pancreatitis score* (HAPS), *Pancreatitis Activity Scoring System* (PASS) and the *Baltharzar-Score* mostly achieved a sensitivity around 70% for early prediction of severe AP [7–12]. Thus, continued efforts are required to investigate reliable biomarkers for the prediction of severity in order to guide early clinical management of AP patients.

There is an emerging body of evidence that the microbiome exerts a crucial impact on the pathophysiology of different pancreatic diseases [13]. Normal pancreas tissue and pancreatic ductal adenocarcinoma (PDAC) harbor distinct compositions of microbiota [14, 15]. Regarding PDAC, the orointestinal and tumoral bacteria and fungi interact with the tumoral immune system, thus influencing progression and overall survival [15–17]. Furthermore, Geller et al. postulated among others that response to chemotherapy depends on the presence of certain bacteria [18]. Using a mouse model Pushalkar et al. demonstrated that bacteria from the oral cavity can migrate into the PDAC [15]. Moreover, a number of cross sectional trials support the hypothesis that oral microbes can be used as a non-invasive diagnostic tool to distinguish PDAC from CP or other premalignant pancreatic lesions such as intraductal papillary mucinous neoplasm (IPMN) [13]. Taken together, there are several recently described associations between PDAC and the microbiome from the oral cavity and the gut.

Only a few studies have focused on the influence of the orointestinal microbiome on inflammatory pancreatic diseases. Due to a systemic inflammatory response syndrome (SIRS) and hypovolemia more than 60% of AP patients suffer from a condition called “leaky gut” [19]. Consequently, circulating microbes can aggravate SIRS. In line with the expected microbiome alterations, two small studies have analyzed feces from AP patients postulating a significantly different intestinal microbiome between healthy volunteers and AP patients, and between mild and severe AP [20, 21].

We aim to comprehensively investigate the orointestinal microbiome as a predictor of course, severity and outcome of patients with AP. In a prospective, multicentric interventional trial we aim to recruit a sufficient number of patients and analyze both the oral and intestinal microbiome by metagenomic and 16S rRNA sequencing.

## Methods: participants, interventions, and outcome

### Study setting

This study is a multicentric prospective study initiated and coordinated from the University Medical Centre Goettingen and supported by the AG Pancreas of the German Society for Digestive and Metabolic Diseases (DGVS) and the European Pancreatic Club (EPC). To date, seven German University Hospitals (LMU Munich, Technical University Munich, Leipzig, Greifswald, Halle, Rostock, Goettingen) and one District Hospital (Hann. Münden) are actively recruiting patients. The principal investigator intends to include more centers across Germany and Europe. All collected samples will be sent to Goettingen where wet-bench and bioinformatical analyses will be conducted. The study was registered 2nd March 2021 (retrospectively) at the US National Library of Medicine at https://www.clinicaltrials.gov (NCT04777812).

### Eligibility criteria

Patients with AP (2 out of 3 following diagnostic criteria: lipase > 3 × of upper limit (> 280U/l), abdominal pain, and imaging modalities (computed tomography (CT), magnetic resonance imaging (MRI) or ultrasound)) indicating AP will be included within 72 h after hospital admission. In our study, amylase elevation will not considered as a diagnostic criterion. In most cases, the first two criteria lead to the diagnosis of AP. Only in rare cases one of the above-mentioned imaging tools will aid to diagnose AP. Typical ultrasound features are: increased volume (> 2.4 cm in pancreas-body diameter), decreased echogenicity and peripancreatic fluid collection [22]. CT and the MRI indicate AP if parenchymal enlargement (diffuse or localized), alterations in density, diffuse margins, acute peripancreatic fluid collection and/or surrounding fat stranding are reported [23]. All recruited patients will sign the informed consent form before buccal and rectal swab collection. Pregnant women, patients < 18 years and patients who are incapable of giving consent will be excluded. The last-mentioned group includes patients who are not authorized to give consent due to psychological or other diseases. Patients who show signs of chronic pancreatitis (CP) on imaging will also be excluded.

The intake of antibiotic and probiotic medication will be recorded before the collection of the swabs and will not be considered as exclusion criteria. Patients will be categorized in 5 groups according to the time of antibiotic medication: current, within the last week, more than 1 week and less than 6 month ago, and more than 6 months ago. Together with other drugs and reported diseases the anti- and probiotic intake will be statistically associated with the orointestinal microbiome patterns and thus treated as potential confounder. Table 1 summarizes medical history and previous medication that is recorded for each patient.

**Table 1:** Medical history and previous medication

Medical history	Previous medication
Cardiovascular diseases (e.g. coronary artery disease, peripheral artery disease, TIA, apoplex)	Metformin
Heart failure	Insulin
Diabetes mellitus	Other antidiabetics
Inflammatory bowel disease	PROTON-pump inhibitors (including occational intake)
Irritable stomach and/or bowel disease	Immune suppressors (including topical immune suppressors affecting intestinal tract)
Clostrioides difficile infection within last year	Antidepressants
Chronic constipation	Neuroleptics
Chronic diarrhea	paracetamol (including occational intake)
Liver cirrhosis	NSAIDs (including occational intake)
Cholestasis	Opiates (including occational intake)
Gastrointestinal malignancy (pancreas, liver and bile duct inclusive)	LAXATIVE (INCLUDING OCCATIONAL INTAKE)
Extraintestinal solid malignancy	Statins
Hematological malignancy	Probiotics
Bariatric surgery	Antibiotics
Other abdominal surgery	
HIV	
Rheumatic disease (arthritis, connective tissue disease, vasculitis)	
Current alcohol use disorder	
Former alcohol use disorder	
Nicotine abusus	

### Outcomes

This prospective translational study aims to evaluate the orointestinal microbiome as a potential biomarker for the course, severity and outcome of patients with AP. From each patient one buccal and rectal swab will be collected within 72 h after hospital admission. As primary endpoint we will analyze the association between microbial composition and the revised Atlanta classification. Secondary endpoints will be the association between microbiome signatures and length of hospital stay, numbers of interventions and mortality. For these analyses, alpha and beta diversity of microbiota will be determined and compared between mild, moderately severe and severe AP. If the analysis will reveal a set of microbes whose presence or abundancies are able to distinguish among the revised Atlanta classification (differential abundances), the microbiome could be employed as early clinical biomarker to guide clinical management of AP patients (e.g. early use of antibiotics).

### Participant timeline

Within 72 h after hospital admission, one buccal and rectal swab will be collected for analysis of the microbiome. Patients will be followed up until their discharge and categorization into mild, moderately severe and severe AP will be performed at the day of discharge.

### Sample size

We calculate to include 400 patients in total (300 with mild pancreatitis (Atlanta I), and 100 with moderately severe or severe AP (Atlanta II–III). This sample size was calculated before enrolment. The sample size is based on the variability of the measured area under the curve (AUC), which is calculated for assessing the predictive accuracy of outcomes. Assuming a true AUC of 0.8, a total of 100 patients per group will provide a power of 85% (99%) when testing against the alternative of an AUC > 0.7 (0.6) at one-sided 2.5% significance level. A 95%-confidence interval for an AUC of 0.8 [0.735; 0.855] will have a total width of 0.12.

### Recruitment

AP patients will be usually treated on gastroenterology or surgical wards. Thus, all participating centers will have access to AP patients on their wards. Furthermore, there is a cooperation between the local principal investigators (PIs) and the emergency room, and PIs will be given immediate notice if patients will have to be directly submitted to IMC or ICU. Therefore, local PIs will be aware of most AP patients admitted to their centers and will be able to enroll them for this study after written informed consent.

## Methods: data collection, management, and analysis

### Data collection methods

**Fig. 1:**
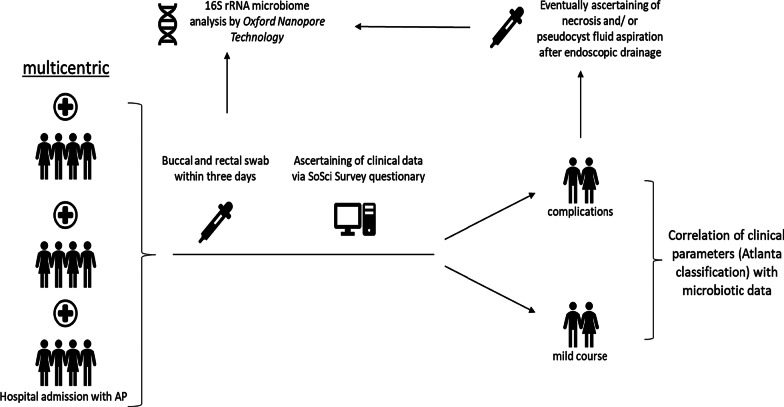
Flowchart of study protocol from enrolment to sample acquisition and correlation of sequencing results with clinical parameters

The oral and intestinal flora will be collected from buccal and rectal swabs. Within three hours after collection swabs will be stored at − 80 °C. External centers will ship samples on dry ice to Goettingen. All wet-bench and bioinformatical analyses will be conducted in Goettingen with an established in-house workflow. First, DNA will be extracted by *PureLink™ Microbiome DNA Purification Kit* (Invitrogen) with a protocol modified according to International Human Microbiome Standards (IHMS). For sequencing Oxford Nanopore Technologies (ONT) MinION and GridION will be employed. ONT represents a method which is considered as the 3rd generation of sequencing. This approach offers two benefits: long reads and a potential real time sequencing. For metagenomic sequencing a minimum of sequencing depth is defined as 10,000 microbial reads for buccal swabs and 25,000 microbial reads for rectal swabs. Regardless of the origin, all 16S rRNA samples need at least 250.000 read counts per sample. These 3 cut-offs were determined by rarefaction curve derived from preliminary experiments. We also developed a bioinformatical pipeline. After basecalling, demultiplexing and trimming the fast5 files (default ONT output) using guppy version 4.4.2, the reads are classified by centrifuge [24]. This tool uses an indexing scheme based on the Burrows-Wheeler transform and the Ferragina–Manzini index. Centrifuge convinces with its high sensitivity though it produces a high rate of false positive reads. To overcome this problem we established an alignment control with minimap2 [25] and a consecutive python script which excludes low quality reads. Both programs are validated for long reads generated by ONT [26]. For the classification with centrifuge and the alignment control with minimap2, a comprehensive library derived from the nucleotide database will be applied containing all complete and incomplete genomes from the National Center for Biotechnology Information (NCBI). Low quality reads will be filtered if they do not pass the following quality criteria: Basecall quality score < 7, centrifuge quality score < 150, alignment score based on Smith Waterman Score < 1000 and an alignment coverage of 50%. Subsequent microbiome analysis such as calculating alpha and beta diversity will be conducted in R-Studio (version 3.6.3). Furthermore, with R the clinical metadata will be correlated to the microbial communities, statistics will be performed, and the graphs will be created. Figure 1 summarizes the study design. All acquired fastq files and the corresponding metadata will be publicly available via Qiita (https://qiita.ucsd.edu/). Before uploading, the human reads will be removed from fastq files using bmtagger.

### Data management

Patient derived metadata will be uploaded after discharging the patient to a privacy protection compliant online database (Sosci-Survey). Once uploaded it will not possible to alter the data. Only the PI from Goettingen will have access to these pseudonymized data.

### Statistical methods

Prior statistical testing, normal distribution and homogeneity of variance will be examined using, QQ-plots and Levene’s test, respectively. If the data will be considered as normally distributed and the homogeneity is not violated, t-test for two groups and ANOVA with consecutive post-hoc tukey-type multiple comparisons for data with more than two groups will be performed. Non-normally distributed variables will be compared with Wilcoxon rank test (two groups) or non-parametric multiple comparisons (Kruskal–Wallis) for more than two groups. Statistical methods will compare groups for primary and secondary outcomes. For alpha diversity the following parameters will be calculated and presented in boxplots: Shannon-Index, Chao1-Estimator and observed richness (i.e. species). For beta diversity the following distances metrics will be performed: Bray–Curtis, unweighted and weighted UniFrac distances. The beta diversity will be visualized with principal coordinate analysis plot (PCoA). Significances will be calculated using PERMANOVA and pairwise adonis test. Further investigation of a significant distance in beta diversity will be conducted with high dimensional class comparisons using linear discriminant analysis of effect size (https://huttenhower.sph.harvard.edu/lefse/). Furthermore, a microbial network will be constructed after centered log-ratio transform of the out-table and visualized with Cytoscape.

Measures of diagnostic and predictive accuracy will be calculated, including area under the curve (AUC) and optimal cut-off point for classification (using simultaneous maximization of sensitivity and specificity).

Cox regression will be performed to interrelate length of hospital stay and the microbiome patterns, which will be calculated and will be most distinctive for the study population. 30-days mortality will also be analyzed using Cox-Regression with a calculated set of microbes as regressors. Number of interventions will be analyzed using negative binomial regression with the same microbes as regressors. Whenever feasible, 95-% confidence intervals will be provided for estimates.

## Ethics and dissemination

### Research ethics approval

This study was reviewed and approved by the Ethics committees of every participating center. As template for the external centers the ethic approval of the Ethic commission of the University Medical Center Goettingen (number: 11/7/19) is applied.

### Protocol amendments

Frequently, (four times a year) all participating centers are updated about the recruitment, and minor or major changes in the study protocol. All changes in the study protocol are also transmitted to the ethic committee.

### Consent or assent

Local PIs obtain written informed consent from potential trial participants. The patients receive a copy of the consent, while the original remains with the local PI.

### Confidentiality

Samples and clinical data are labelled with pseudonyms in every center. Lists with patient´s names and the corresponding pseudonyms remain in each center and only local investigators have access to these sensitive data.

### Access to data

After uploading the metadata to SoSci Survey only the PI from Goettingen has access to the data. All sequencing experiments and data analysis will be conducted in Goettingen. All data remain in Goettingen until publication.

### Dissemination policy

The results of this study will be published in an open-source journal and presented regularly in national and international conferences such as the German Pancreatic Club, the European Pancreatic Club, United European Gastroenterology Week and the annual DGVS meeting.

## Discussion

Early prediction of the course of AP remains challenging. We hypothesize that the orointestinal microbiome can be a potential biomarker for the course, severity and outcome of patients with AP. In line with our hypothesis, two small single-center studies from China detected alterations of fecal microbes which could distinguish between different grades of severities and between healthy volunteers and AP patients [20, 21]. With this prospective multicentric study we aim to investigate the microbiome in AP patients in a highly standardized and statistically powerful setting. To date, 8 centers are actively enrolling patients in P-MAPS. We intend to further expand the trial in Germany and involve large European centers. We not only focus on the intestinal microbiome but also analyze oral communities. There is emerging evidence that the oral flora plays a crucial role in pancreatic diseases, too [13]. In contrast to the existing data from China, we collect microbiome samples from rectal swabs but not from stool samples. Due to an oxygen gradient, there are different niches for microbes in the gut [27]. The microbial composition found in the lumen of stool fluctuates more frequently and harbors mostly anaerobic bacteria, whereas mucosa adherent microbes are often aerotolerant, more stable over time, and directly interact with the gut immune system [28, 29]. Thus, rectal swabs yield similar microbial communities as biopsies obtained by colonoscopy [30–33]. The longitudinal intraindividual stability of the microbial composition and the sufficient yield of biomass was confirmed before selecting rectal swabs as appropriate sampling method for the intestinal microbiome [31–33]. Therefore, it is widely accepted that rectal swabs are convenient and reliable alternatives to invasive biopsies.

Moreover, P-MAPS will rely on metagenomic sequencing which will allow a more profound analysis of the microbiome of AP patients for the first time. Prior to the initiation of this study, comprehensive wet-bench protocols and a bioinformatical pipeline were established allowing an accurate microbiome analysis sequenced with ONT. In analogy to the workflow published by Sanderson et al. [26], validated programs were combined and further adjusted with an inhouse python script [24, 25]. All scripts (python, R, bash) and protocols will be publicly available with publishing the data. With the combination of rapid library preparation and real time sequencing ONT enables an insight into microbial composition within a few hours. The method could be easily transferred to the daily clinical workflow of AP patients.

In conclusion, this prospective multicentric study aims to analyze the orointestinal microbiome of AP patients and evaluates both microbial communities as a potential biomarker for the course, severity and outcome of patients with AP.

## Data Availability

The datasets used and/or analysed during the current study are available from the corresponding author on reasonable request.

## Abbreviations

AP: Acute pancreatitis
AUC: Area under the curve
CT: Computer tomography
IPMN: Intraductal papillary mucinous neoplasm
MRI: Magnetic resonance imaging
NGS: Next Generation of Sequencing
ONT: Oxford Nanopore Technologies
PDAC: Pancreatic ductal Adenocarcinoma
PI: Principal investigator
P-MAPS: Microbiome as predictor of severity in acute pancreatitis
